# The viral prescription pad - a mixed methods study to determine the need for and utility of an educational tool for antimicrobial stewardship in primary health care

**DOI:** 10.1186/s12875-020-01114-z

**Published:** 2020-02-22

**Authors:** Christine Lee, Maryam Jafari, Regan Brownbridge, Casey Phillips, Jason R. Vanstone

**Affiliations:** 1 College of Pharmacy and Nutrition, University of Saskatchewan, 104 Clinic Place, Saskatoon, SK S7N 2Z4 Canada; 2 Dr. T. Bhanu Prasad Medical Professional Corporation, 3401B Pasqua St., Regina, SK S4S 7K9 Canada; 3 College of Medicine, University of Saskatchewan, 107 Wiggins Rd., Saskatoon, SK S7N 5E5 Canada; 4 Antimicrobial Stewardship Program, Saskatchewan Health Authority – Regina Area, 4B35, 1440 – 14th Ave., Regina, SK S4P 0W5 Canada; 5 Stewardship and Clinical Appropriateness, Saskatchewan Health Authority – Regina Area, 4B35, 1440 – 14th Ave., Regina, SK S4P 0W5 Canada

**Keywords:** Antimicrobial stewardship, Primary health care, Patient education, Viral prescription pad

## Abstract

**Background:**

In order to combat rising rates of antimicrobial resistant infections, it is vital that antimicrobial stewardship become embedded in primary health care (PHC). Despite the high use of antimicrobials in PHC settings, there is a lack of data regarding the integration of antimicrobial stewardship programs (ASP) in non-hospital settings. Our research aimed to determine which antimicrobial stewardship interventions are optimal to introduce into PHC clinics beginning to engage with an ASP, as well as how to optimize those interventions. This work became focused specifically around management of viral upper respiratory tract infections (URTIs), as these infections are one of the main sources of inappropriate antibiotic use.

**Methods:**

This mixed methods study of sequential explanatory design was developed through three research projects over 3 years in Regina, Saskatchewan, Canada. First, a survey of PHC providers was performed to determine their perceived needs from a PHC-based ASP. From this work, a “viral prescription pad” was developed to provide a tool to help PHC providers engage in patient education regarding appropriate antimicrobial use, specifically for URTIs. Next, interviews were performed with family physicians to discuss their perceived utility of this tool. Finally, we performed a public survey to determine preferences for the medium by which information is received regarding symptom management for viral URTIs.

**Results:**

The majority of PHC providers responding to the initial survey indicated they were improperly equipped with tools to aid in promoting conversations with patients and providing education about the appropriate use of antimicrobials. Following dissemination of the viral prescription pad and semi-structured interviews with family physicians, the viral prescription pad was deemed to be a useful educational tool. However, about half of the physicians interviewed indicated they did not actually provide a viral prescription to patients when providing advice on symptom management for viral URTIs. When asked about their preferences, 76% of respondents to the public survey indicated they would prefer to receive written or a combination of verbal and written information in this circumstance.

**Conclusions:**

PHC providers indicated a need for educational tools to promote conversations with patients and provide education about the appropriate use of antimicrobials. Viral prescription pads were regarded by family physicians and patients as useful tools in facilitating discussion on the appropriate use of antimicrobials. PHC providers should exercise caution in opting out of providing written forms of information, as many respondents to the general public survey indicated their preference in receiving both verbal and written information.

## Background

In 2016, the United Nations declared antimicrobial resistance a health issue of global concern [1]. Globally, more than 700, 000 people die each year from antimicrobial resistant infections and this number could rise to more than 10 million by 2050 [2]. Development of antimicrobial resistance is driven by our use of antimicrobials in humans, animals, and the environment. In Canada, more than 92% of antimicrobial prescriptions are dispensed from community pharmacies each year and Saskatchewan is the second highest user of community-prescribed antimicrobials in Canada [3]. These data indicate a need for antimicrobial stewardship in the primary health care (PHC) setting. However, because the majority of antimicrobial stewardship programs (ASP) operate in hospitals, there is a relative paucity of information about effective stewardship strategies in PHC (see, for example, [4]). Even less is known about what the broad range of PHC providers expect from an ASP, particularly within Canada (see, for example, [5–7]).

Despite the majority of ASP research coming from the acute care setting, there is some evidence for effective antimicrobial stewardship strategies in PHC. Bozella et al., for example, reviewed a number of studies providing evidence-based strategies to improve antibiotic prescribing in ambulatory care settings [8]. However, it should be noted that it can be difficult to implement some of these strategies (e.g., clinician education, audit and feedback, and communication training, implementing algorithms, and prescription justification) based on the resources required to do so. Smaller, resource-limited ASPs, like our local ASP, do not necessarily have the human resources, access to data, and technical capabilities to undertake all of these types of initiatives. Patient education (e.g., via the use of handouts) about appropriate antibiotic use has also been studied and there is evidence to indicate that including written information as part of patient education about appropriate antibiotic use may help to reduce antibiotic prescribing [9].

Thus, we set out to determine, with feedback from local PHC practitioners, what types of initiatives are both practical and feasible for a local ASP to integrate into PHC clinics. In response to the perceived needs of local PHC providers, the local ASP for the Regina Area of the Saskatchewan Health Authority (formerly the Regina Qu’Appelle Health region; based in Regina, Saskatchewan, Canada) developed a “viral prescription pad” (Sup. Fig. 1) to be used as a tool during consults with patients suffering from viral infections. The viral prescription pad focuses particularly on upper respiratory tract infections (URTI; i.e., bronchitis, acute otitis media, pharyngitis/tonsillitis, rhinitis, and sinusitis). As the majority (90%) of URTIs are viral in etiology, that makes this is an important group of infections for antimicrobial stewardship because they represent some of the most common conditions with unnecessary use of antibiotics [10, 11]. This tool can help guide a provider through a consult and it provides documentation of non-antibiotic treatment options for patients. The viral prescription pad developed by the local ASP has been adopted with minor modifications by other organizations both provincially and nationally [12–14].

The series of studies described herein were undertaken with the overall aim of guiding the development and implementation of a PHC-based ASP, including: 1) understanding the perceptions of PHC providers about what they believe is required for a PHC-based ASP, 2) understanding the perceived utility of tools (e.g., viral prescription pad) developed by the local ASP, and 3) understanding the public’s perception about the best medium (i.e., verbal or written) by which to receive the information contained in the viral prescription pad.

## Methods

This was a mixed methods study of sequential explanatory design [15], a methodology chosen because of the nature of the investigations (i.e., the initial survey provided quantitative information which informed the qualitative interviews that followed). The study was developed through three research projects conducted in Regina, Saskatchewan, Canada over 3 years (May 2016 – April 2019). As such, not all details are presented for each project; instead, we focus on specific aspects of each project which led from one to the next. For example, while multiple educational tools were developed following the initial PHC provider survey, our focus in this manuscript is on the viral prescription pad, as it was indicated to be the tool most frequently used by clinicians. All figures were prepared using Tableau Desktop v9.0 software (Seattle, USA).

### PHC provider survey (May – August 2016)

The PHC provider survey was completed to inform the development and implementation of ASP initiatives in the local PHC setting. A link to an online survey (www.fluidsurveys.com) was distributed to family physicians (*n* = 217) and nurse practitioners (*n* = 40) in the former Regina Qu’Appelle Health Region, as well as community pharmacists (*n* = 1109) and dentists (*n* = 487) throughout Saskatchewan. These professional groups were chosen as they represent PHC practitioners who are prescribers and/or play a role in dispensing antimicrobials to patients (e.g., pharmacists). The link was sent via email lists held by respective departments, professional associations, or regulatory bodies along with an introductory letter from the research team. There were a minority of family physicians without an email contact, to whom the survey was faxed, instead. Due to the limited availability of members of the research team, the survey was open for a period of 8 weeks. A reminder email/fax was sent midway through the survey period.

Surveys were composed of 16–19 questions (depending on the respondents’ specialty) and consisted of 5-point Likert scale, sliding bar, and multiple choice questions (Sup. Fig. 2). The survey was developed by the research team (CL, CP, and JRV) based on previously published studies (e.g., [16–19]) and with input from relevant professionals about the appropriateness and comprehension of questions. Descriptive statistics were used for analysis and responses to Likert-type questions were categorized into Agree (Strongly Agree or Agree) and Disagree/Neutral (Neutral, Disagree, or Strongly Disagree).

### Viral prescription pad

Following completion of the PHC provider survey, the viral prescription pad (Sup. Fig. 1) was developed as a tool to aid in educating both providers and patients about appropriate use of antimicrobials, particularly for URTIs. The prescription pad was developed by the research team (CL, CP, and JRV) with input from clinicians (physicians, pharmacists, and nurse practitioners) and patient advisors working with the local ASP. The informational content was selected by drawing from examples of viral prescription pads which already existed (i.e., were available online) and information pamphlets which were produced within the health region. All content was assessed for medical accuracy by relevant clinicians. The intent was to create a prescription pad with greater appeal to end-users (e.g., larger size, colour document, accessible language, personalized to the patient, etc.). Distribution of the prescription pad was aided by the local PHC departmental staff who delivered printed pads to local PHC clinics (*n* ≈ 50) along with written instructions on the intended use of the tool. It was also made available online via the local ASP website and was integrated into some PHC clinic electronic medical record systems.

### Physician interviews (November 2017 – May 2018)

Following the PHC provider survey and ensuing development and dissemination of the viral prescription pad, we performed interviews with 12 family physicians to better understand their perceptions of the utility of this tool. An email was sent to family physicians in the former Regina Qu’Appelle Health Region (n ≈ 200) to recruit participants. We also reached out directly to “physician champions” (i.e., physician leaders we had engaged with previously) to help with recruitment. Unfortunately, we experienced difficulty recruiting physicians for interviews which limited us to 12 participants; however, as the interviews were analyzed, it was determined that we had reached saturation as there were no new themes emerging. Additionally, Guest et al. provide evidence that 12 interviews may be enough to reach saturation for a relatively homogenous population [20], which we had in our participants. Due to a lag in procuring funding for a research assistant, interviews began approximately 12 months following the launch of the viral prescription pad and took approximately 4 months to complete (i.e., to schedule and complete all 12 physician interviews). Participants completed a written informed consent document prior to the beginning of their interview. Face-to-face or over-the-phone in-depth, semi-structured interviews were performed using an interview guidance script (Sup. Fig. 3).

Interviews were conducted by MJ between December 2017 and March 2018. The interviewer had not previously worked with the ASP and had no other connections to the development of the viral prescription pad. This was clearly outlined to potential participants in the initial contact letter that was sent to physicians when requesting an interview. We believe this provided an opportunity for interviewees to freely express their feelings and perspectives in a one-on-one setting. Interviews lasted 20–40 min and an audio recording was made and electronically transcribed by the interviewer. Transcripts were randomly assigned a study identification code allowing interviewees’ remarks to remain anonymous for data analysis and reporting. A thematic analysis approach was used to analyse the qualitative data [21]. The analysis was performed by three researchers (MJ, JRV, and a research intern) who each read the transcripts independently. Themes and subthemes were identified and compared until consensus was reached. Saturation occurred when no new themes were discovered.

The interviews allowed us to determine if and how the viral prescription pads were being used in practice by physicians, and if there were any suggestions on how to improve this educational tool. One prominent theme that arose in this study was the question of the best medium with which to provide health care information to patients (e.g., verbal communication, printed literature, videos, etc.). This question of the optimal medium of communication led to the final research project.

### Public survey (November 2018 – April 2019)

An online public survey was conducted to determine preferences for receiving information from PHC providers (verbal, written, or a combination of both) about symptom management for viral URTIs. The survey was developed by the research team (RB and JRV) with input from the local PHC network managers and patient advisors. The survey was created using the Research Electronic Database Capture (REDCap) web-based software [22, 23] and disseminated to local PHC clinics via an advertisement poster to be displayed in waiting rooms. The online survey link was also shared through social media (Facebook, Twitter, and LinkedIn) via personal accounts of the researchers (there was no paid advertising). Once again, due to limited availability of members of the research team, the survey was open for a period of 6 weeks. Reminders were posted to social media midway through the survey period. To further encourage participation, respondents had an opportunity to receive a gift card (10 cards worth CAD 20 each) after completing the survey.

This survey consisted mainly of multiple choice type questions with Likert scales for responses (Sup. Fig. 4). Respondents consented by completing the survey and were asked to provide some basic demographics (age, sex, and highest educational level achieved) which were used to stratify the data during analysis. Descriptive statistics were used for analysis.

To simplify the survey, it was focused around a visit in which the patient would be seeking care for an URTI determined to be viral by the provider. The survey asked if the respondent would be satisfied with receiving verbal instructions alone, or if they would prefer a physical handout to be able to refer to after their visit is complete. Furthermore, we asked if they found our example of a viral prescription pad to be a beneficial tool.

## Results

### PHC provider survey

**Fig. 1:**
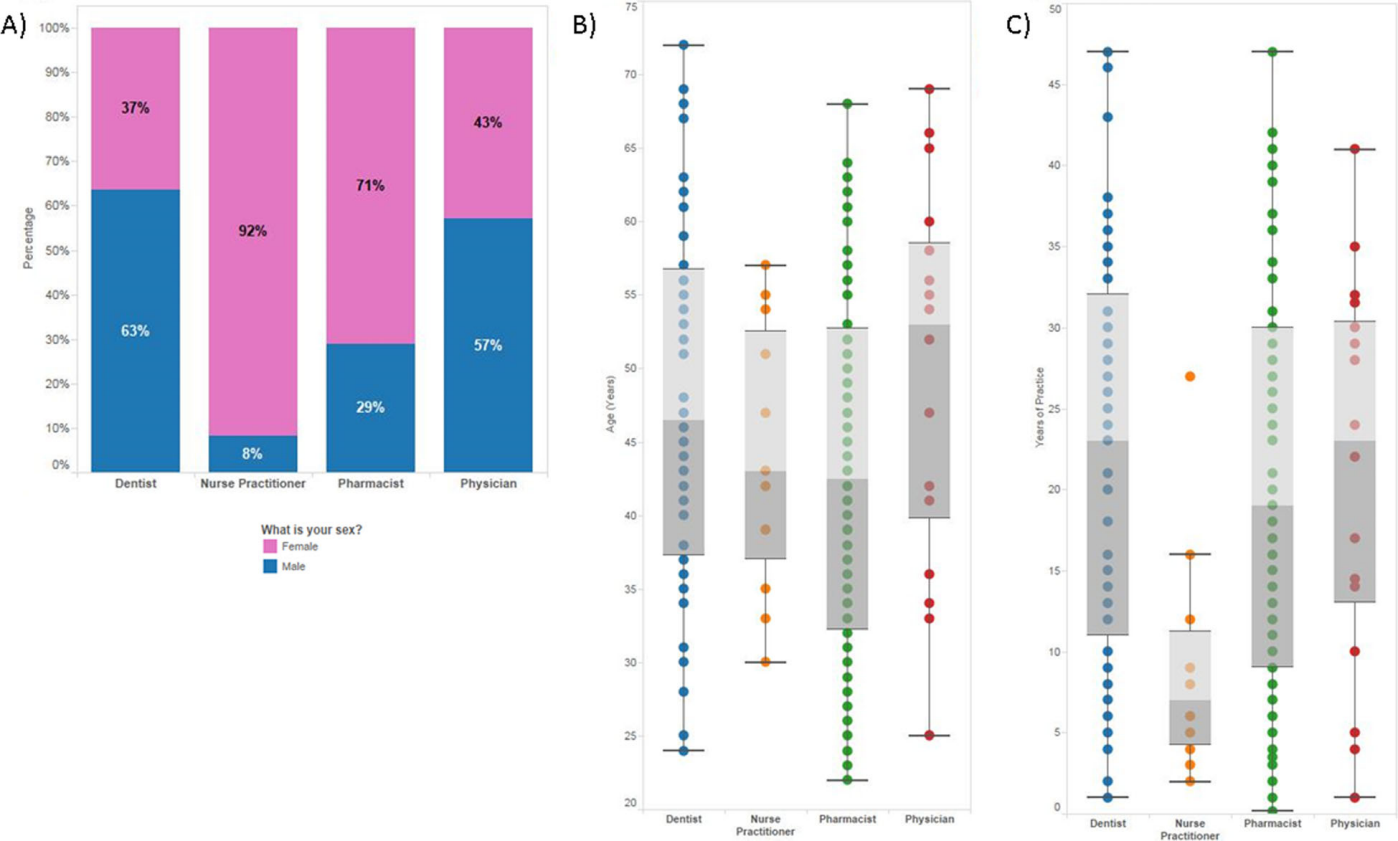
Health care provider demographics. **a** Percentage of respondents who identified as male or female in each health care provider category. **b** Box plot indicating the age of respondents in each health care provider category. **c** Box plot indicating the years of practice of respondents in each health care provider category

Responses were received from 234/1855 (13%) survey invitations that were sent to potential participants. This included 21/219 (10%) family physicians, 12/40 (30%) nurse practitioners, 138/1109 (12%) community pharmacists, and 63/487 (13%) dentists. The demographics of respondents are shown in Fig. 1. Dentists and physicians had the highest rate of male respondents (63 and 57%, respectively), while nurse practitioners and pharmacists were primarily female (92, and 71%, respectively) (Fig. 1a). The median age of respondents varied between groups of health care providers (Fig. 1b), with pharmacists and nurse practitioners being the lowest (43 years) and physicians being the highest (53 years). The median years of practice also varied between groups of health care providers (Fig. 1c), with nurse practitioners being the lowest (7 years) and physicians and dentists being the highest (23 years).

**Fig. 2:**
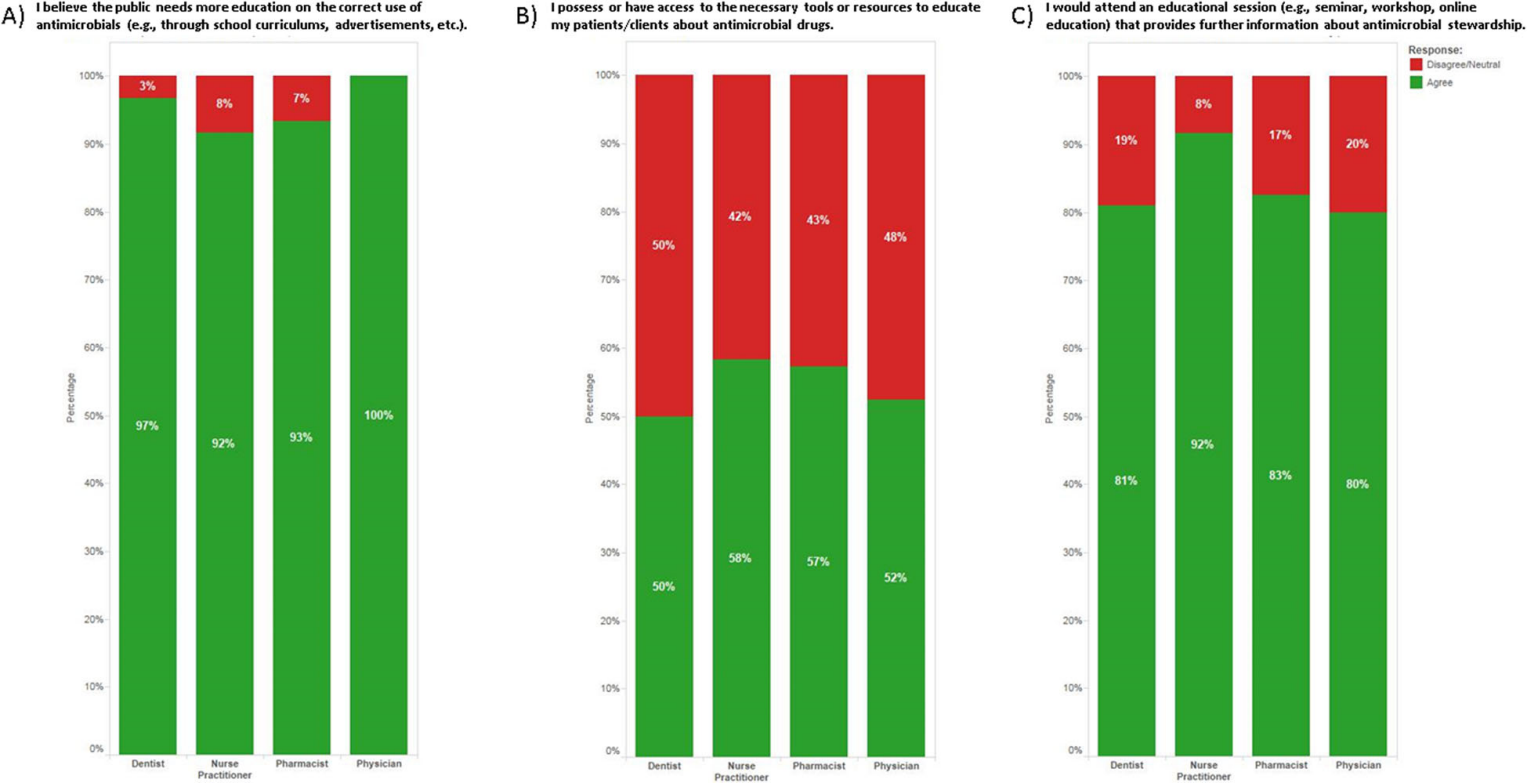
Responses to questions about antimicrobial stewardship educational tools and resources. **a** More than 92% of respondents in each category of health care provider agreed with the statement, “I believe the public needs more education on the correct use of antimicrobials (e.g. through school curriculums, advertisements, etc.).” **b** Less than 58% of respondents in each category agreed with the statement, “I possess or have access to the necessary tools or resources to educate my patients about antimicrobial drugs.” **c** More than 80% of respondents in each category agreed with the statement, “I would attend an educational session (e.g., seminar, workshop, online education) that providers further information about antimicrobial stewardship”

The set of questions most relevant to this manuscript involved understanding the perceptions of health care providers around antimicrobial stewardship education and related resources (Fig. 2). When asked if they agreed with the statement, “I believe the public needs more education on the correct use of antimicrobials,” more than 92% of respondents in each health care provider category agreed (Fig. 2a). When asked if they agreed with the statement, “I possess or have access to the necessary tools or resources to educate my patients about antimicrobial drugs,” less than 58% of each category of respondents agreed (Fig. 2b). Finally, when asked if they agreed with the statement, “I would attend an educational session (e.g., seminar, workshop, online education) that provides further information about antimicrobial stewardship,” more than 80% of respondents in each category agreed (Fig. 2c).

The results of the survey led to the creation of the viral prescription pad to be used as a tool for educating prescribers and patients about the appropriate use of antimicrobials, particularly for viral URTIs. This tool was one focus of the follow-up study in which physicians were interviewed about the perceived utility of a viral prescription pad in practice.

### Physician interviews

Participants for the physician interviews were recruited from one rural and two urban clinics, all of which were concurrently involved in an audit and feedback initiative with the local ASP. Of the 12 physicians, 11 (92%) practiced in urban clinics and 7 (58%) practiced in an academically-affiliated clinic. The model of reimbursement for all physicians was salary-based or daily-based payments.

Overall, physicians were in favour of using educational tools to promote conversations with their patients about appropriate antimicrobial use. For this manuscript, the focus will be on the comments around the viral prescription pad that was developed by the local ASP following the PHC provider survey. Physician opinions on when to use the viral prescription pad were mixed; some reported they had frequently used it during consultations and found it very useful, while others stated they preferred to communicate verbally, without handing the patient any written information. Some physicians stated they only used it in cases where they were in disagreement with a patient regarding the prescription of an antibiotic.

“I think there's nothing I don't like about the [viral prescription] pad. It does help. I use it for 50% of my patients. So, for those who have already been to one clinic and then are coming to see me, and I feel they need something to be convinced. Maybe the other doctor has told them but they still need that second opinion. So those are the ones that I use the pad for.” (Participant D)

Those who used the viral prescription pad stated they used it as an opportunity for educating patients about antibiotic resistance. They believed they have seen less resistance from patients when not prescribing unnecessary antibiotics. Because the pad lists the most common viral infections with the duration of symptoms, patients are more likely to be convinced they have a viral infection when they fit into one or more categories. Physicians particularly liked that the pad mentioned the duration of symptoms, so that patients know it is normal to have a cough, for example, for several weeks after an infection.

“I have seen a very good and positive response with the use of just these handouts [viral prescription pads]. I have noticed the difference between just telling them that it's a viral infection and why I'm not giving them antibiotics, and with the use of this handout. I find greater and easier acceptance when I explain [to my patients] using this. It doesn't just say it’s a viral infection. It includes this whole gamut of symptoms that patients come with. So, every patient that I'm going to talk to about these would fit into one or the other or sometimes even two or more of these categories. I think that is one of the very good features which hits the take home message very easily.” (Participant S)

“At least it's helping to limit the number of antibiotics. The thing is, as a doctor, whether it [viral prescription pad] is there or not, if it [antibiotic] is not indicated it is not indicated. So, even if this [viral prescription pad] wasn't there you would still not prescribe antibiotics if it wasn't indicated. So, the only thing I would say is it helps the patient to understand better the reason why you are not doing it [prescribing antibiotics]. So, overall it is actually helping … It’s more about patient awareness. It increases awareness for them about using antibiotics when they’re not indicated.” (Participant W)

Physicians who used the viral prescription pad believed that it guides an appropriate consultation (as it is comprehensive), supports or reinforces physician’s explanations, improves communication, acknowledges the physician’s empathy about the patient’s health condition, optimizes patient reassurance during a consultation, and can be customized to each patient (space is available for physicians to add extra comments or instructions).

“I would say that overall the response is pretty positive. I just find, since I started using it [viral prescription pad] and taking the approach using this, and explaining things by using the pad, I've had a lot less resistance and a lot less kind of difficult conversations with patients around expectations. I feel like it helps smooth the conversation out about what is the appropriate thing to do in this situation. I don't know, maybe it's just my approach is improving or what, but it seems like I’m having less of those tricky conversations.” (Participant B)

“We can write specific instructions to them, so that they know that we're not just dismissing their symptoms but also, you know, we recognize that they're having a tough time but they don't need any antibiotics. Here’s some things that you can do and here's some reasons to come back to see me. I think it’s great.” (Participant B)

“I really like it. I can’t mention anything as a negative point … I use them for at least more than 80% of my patients. It's a very good support for all the explanation a physician provides with the patient … you know, the patient expectation about the symptoms and the duration. And you know they usually agree that they should wait for minimum seven days, ten days, two weeks for the symptom to get better by itself.” (Participant N)

Physicians in favor of using the pad also mentioned that by providing patients with evidence-based, tangible advice in the style of a prescription, patients would not feel that they did not receive any kind of care. In addition, most of them felt the pad is self-explanatory and uses an appropriate level of language.

“It gives them something, something concrete that they can look at and refer to. So, I think they don't feel empty handed … ” (Participant B)

“So, the patient is walking away with something. It’s not just the patient coming in and you say no it's a virus, you go home. I think they feel more like they actually came to see a doctor. You give them this and everything is filled out and they can look at it and see that is coming from Regina Qu*'*Appelle Health Region. So, it’s not just the doctor telling me not to use it [an antibiotic].” (Participant W)

“Sometimes people leave the office and they don't remember everything that you said but if they have the prescription, they're able to refer to it. I like the idea that patients leave us with something that's informative and in the style of a prescription.” (Participant C)

The majority of physicians in the academically-affiliated clinic didn’t use the viral prescription pad. According to many of the physicians in the clinic, family medicine residents would probably use it, as they often provide direct patient care and education. Most of the physicians who did not use the viral prescription pad still viewed it as a good educational tool (comprehensive and evidence-based). Major reasons for not using the tool were due to years of experience, continuity of care, and establishing trust/a good doctor-patient relationship. They stated they would verbally reassure patients there was no need for antibiotic use without any need to hand out written material. Besides this, they would intuitively explain the content of the viral prescription pad to their patients. As such, they felt using the pad wouldn’t add to their approach. Some physicians stated this tool would possibly be useful for newer providers, as they might not have the symptoms and treatment options readily available.

“I have been in practice for many years. I know my patients very well. I have probably unusual continuity with my patients compared to what the system is like now, where you see a different doctor all the time. I don't feel like my patients need that [viral prescription pad]. They just need my verbal reassurance that they don't need an antibiotic … I understand the rationale for having that viral prescription pad. But to me is sort of like saying, oh everybody needs a piece of paper and a prescription to walk out of a doctor's office. I'm trying to go one step further and say you don't need anything except reassurance that this is viral and symptomatic treatment will do … . I know the residents use it. But they don't have continuity with the patient, I do. They're trying to give an official treatment.” (Participant J)

“I usually tell patients this information verbally. And I don't know how much this [viral prescription pad] really adds, to be honest, to what I already tell them.” (Participant P)

The results of the physician interviews led to the concern that some physicians were opting not to use the viral prescription pad as intended (i.e., to be handed to a patient with an explanation of why antibiotics were unnecessary and how to manage their URTI symptoms). In order to determine if patients preferred to have a written handout instead of, or in addition to, verbal instructions for URTI symptom management, we performed a follow-up public survey.

### Public survey

**Fig. 3:**
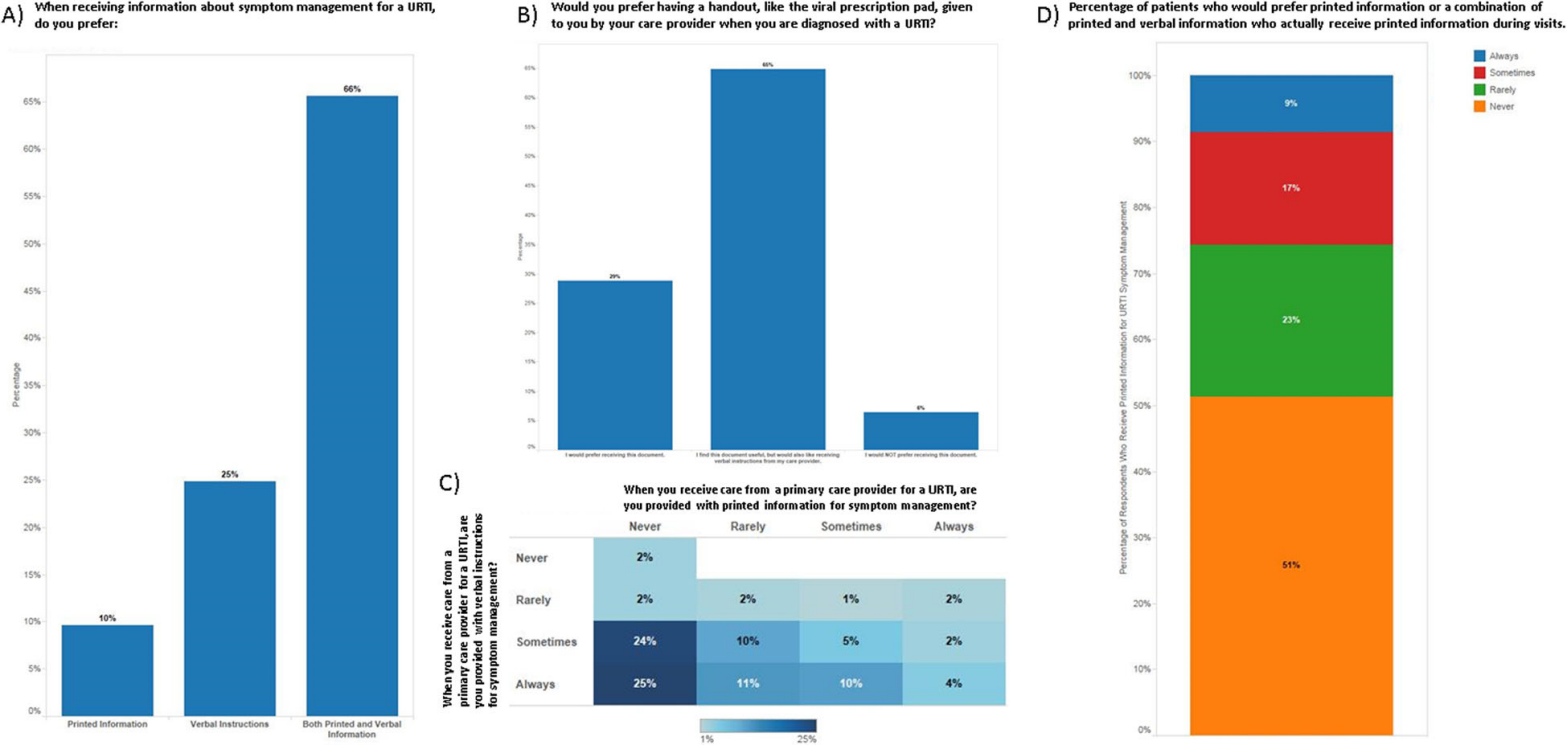
Responses to questions about patient preference for information delivery regarding symptom management for upper respiratory tract infections (URTI). **a** 66% of respondents indicated they would prefer to receive both printed and verbal information about symptom management for a URTI. **b** 65% of respondents indicated they would prefer a handout like the viral prescription pad to be provided when they are diagnosed with a URTI, along with verbal instructions. **c** 49% of patients are sometimes or always provided with verbal instructions, but are never provided with printed information for symptom management for a URTI. Only 21% of respondents indicated that they are sometimes or always provided both written and verbal instructions. **d** Of the patients who indicated they would prefer to receive printed information or both printed and verbal information for URTI symptom management, 74% indicated they rarely or never receive printed information during their visits

Respondent demographics for the public survey are provided in Table 1. Of the 125 respondents, 99 (79%) were female, the median age was 35 (range: 21–70), 116 (93%) had a family doctor, and 111 (89%) had completed post-secondary or graduate level degrees. For this manuscript, the responses relevant to patient preferences for receiving printed information for the treatment of URTIs are presented (Fig. 3). Approximately ^2^/_3_ of respondents indicated that they prefer receiving both printed and verbal information from their care providers with respect to symptom management for a URTI (66%, Fig. 3a) and that they like the viral prescription pad, but also would like to receive verbal instructions along with it (65%, Fig. 3b). When combining responses to the questions of how often respondents receive either printed information or verbal instructions for symptom management, only 21% indicated they sometimes or always receive both forms of information (Fig. 3c). Most respondents (70%) indicated that, while they sometimes or always receive verbal instructions, they rarely or never receive printed information (Fig. 3c). When combining respondents who indicated they would prefer either printed information or a combination of printed information and verbal instructions for symptom management, 74% of these respondents indicated they rarely or never receive printed information (Fig. 3d).

**Table 1:** Respondent demographics for the public survey

Category	Sub-Category	N (%)
Sex	Male	26 (21)
	Female	99 (79)
Physician Status	I have a family doctor.	116 (93)
	I generally use a walk-in/emergency room.	9 (7)
Highest Level of Education Completed	Elementary	1 (< 1)
	High School	13 (11)
	Post-Secondary	79 (63)
	Graduate	32 (26)
Median Age (Range)		35 (21–70)

Overall, 29% (36/125) of respondents indicated they expect to receive antibiotics from their primary care provider to treat an URTI. Of note, of the 45% (56/125) of respondents who indicated that they sometimes or often seek care for symptoms of an URTI, 41% (23/56) indicated they also expect to receive antibiotics. Respondents’ perceived knowledge of antibiotics also correlated with their expectations to receive antibiotics for the treatment of an URTI (i.e., patients with the lowest reported understanding of antibiotics and lower levels of education often indicated they expect antibiotics for an URTI, and vice versa). Only 28% (33/120) of respondents who rated themselves as having a moderate, good, or high level understanding of antibiotics indicated they would expect to receive an antibiotic to treat an URTI. However, 60% (3/5) of respondents who indicated a minimal understanding of antibiotics also indicated they would expect to receive an antibiotic to treat an URTI.

Thus, these three studies create a narrative arc wherein the initial study, which was developed to better understand PHC practitioner needs from an ASP, indicated a need for educational tools. Following the development and dissemination of the tools (particularly the viral prescription pad), the second study determined the perceived utility of the prescription pad through physician interviews. While there was consensus that the prescription pad is useful for educating patients about appropriate antimicrobial use, there was also evidence that some physicians were not using the prescription pad as intended and were opting to provide only verbal advice for viral URTI symptom management. Hence, the third study provided evidence that patients prefer to receive both written and verbal instructions for symptom management of viral URTIs.

## Discussion

### PHC provider survey and educational tool development

For this study, a broad approach was initially taken to include PHC providers not traditionally surveyed in the literature (i.e., nurse practitioners and dentists in addition to physicians and pharmacists). This approach ensured that the developing local ASP would represent the perceived needs of all providers who would need to be engaged. The high proportion of PHC provider survey respondents who indicated they believed there was a need for more tools for patient education provided the local ASP with the incentive to develop resources to be made available for this purpose. We searched the available literature and collected or created documents (including informational pamphlets and documents such as the viral prescription pad, Sup. Fig. 1) which were shared with health care providers through multiple channels, including a program website, in-person visits to community clinics, long-term care facilities, and tertiary hospitals, and through various local newsletters to different clinician groups.

### Physician interviews

Approximately 12–16 months following dissemination of the educational tools and resources, we performed interviews with community-based family physicians to better understand if and how they were being used. The viral prescription pad was the most frequently used resource for patient education. Physicians who used this resource felt they experienced fewer challenges convincing patients that antibiotics were not necessary and found it helped guide conversations and optimized patient reassurance as it clearly lists the realistic recovery time, self-management approaches for symptom relief, and return to care indications. This is consistent with other studies that indicate professional medical advice positively impacts patients’ perceptions and attitude towards their perceived need for antibiotics, particularly when they are advised on what to expect during the illness, including the duration of disease and self-management strategies [24]. This tool also helps to engage patients in person-to-person communication, which is key for educating them about unnecessary use of antibiotics. These findings are consistent with studies that highlight the importance of the clinician-patient (or parent) interaction in managing illnesses; patient/parent satisfaction depends more on effective communication than receiving an antibiotic prescription [25, 26]. This tool could be particularly useful in very busy or walk-in clinics to increase communication and decrease the likelihood of resistance from patients who are expecting an antibiotic. Some studies suggest patient information leaflets encourage patients to raise concerns and discuss health related issues during the consultation which can increase patient satisfaction and their perception of communication, particularly for short consultations [10].

Interestingly, for those physicians who reported not using the viral prescription pad, one of the reasons was due to the fact that they felt they already had well established doctor-patient relationships and the viral prescription pad did not provide any additional benefit. There are numerous studies examining the question of the best medium by which to provide information to patients in various acute care settings (e.g., oncology, surgery, chronic disease [27–31];). Whether it’s better to provide patients with literature to inform and educate (e.g., about management of a chronic condition or preparation for a procedure) or if verbal instructions from health care providers are sufficient appears to be somewhat dependent on the specifics of the health problem and the health literacy of the patient population. Watson and McKinstry reviewed interventions to improve recall of medical advice in health care consultations and found that, while written and audio recorded instructions seem to improve recall in most cases, few interventions use psychological models of recall in their design [32], making it difficult to generalize these findings. Furthermore, as we move into an age of personalized medicine, it may also be important to reflect on individual differences in learning when trying to determine the best method for delivering information [33].

Some studies have found written information to be beneficial [34] and others have found a combination of both verbal and written information to be ideal [24, 35, 36]. It is important for care providers to reassure patients that their viral illness will not benefit from the use of antibiotics and provide them with advice on symptom relief. Verbal communication between the care provider and patient is one way of providing this information. However, this is not always done in an ideal fashion and much research has gone into determining optimal ways to verbally communicate with patients [37–39]. Based on the interviews that were performed, nearly half of the physicians indicated they prefer to provide only verbal symptom management information to their patients with URTIs.

### Public survey

With that information in hand, a follow-up public survey was performed to determine preferences for receiving information (verbal or printed) as it relates to symptom management for URTIs. In our survey, 93% of respondents indicated they had a family doctor (Table 1) and 76% indicated they would prefer written or a combination of verbal and written instructions for URTI symptom management (Fig. 3a). This indicates that, even with the potential for an established doctor-patient relationship, many patients may still prefer receiving written information when it comes to symptom management for URTIs.

The results of the public survey share similarities with previously published results. Gaarslev et al. [40] found that 19.5% of respondents to their patient survey expected physicians to prescribe antibiotics for a cold or flu; this number was similarly low in our cohort of respondents (29%). Although conclusions could not be drawn based on the level of education of respondents as per Gaarslev et al. (due to the low number of respondents with a high school education or lower), it was found that a larger proportion of respondents with a lower reported understanding of antibiotics indicated they expect antibiotics for URTI treatment (60% with minimal understanding of antibiotics versus 28% with moderate or better understanding).

The three projects described herein have allowed us to develop and implement an educational tool for antimicrobial stewardship in PHC practices, with evidence to support its use. As the local ASP developed, we used the initial survey to reach out to PHC practitioners for their input on what is needed for an ASP to be embedded in PHC. From this work, it was determined that there was a need for educational tools and the viral prescription pad was developed. To better understand its perceived utility, we then interviewed family physicians. The information from these interviews provided evidence that practitioners do indeed find this to be a useful tool, although it wasn’t always being used as intended. Thus, our third project set out to determine if patients prefer to receive verbal, written, or both verbal and written instructions when being provided information about viral URTI symptom management.

### Strengths and limitations

Due to the response rates for the different health care provider categories in the PHC provider survey being below the optimal sample size, the power of this study is limited, along with the ability to generalize the results. However, for the data presented herein, there is a high level of congruency among the respondents’ answers (Fig. 2), indicating agreement on the need for educational tools. This study is also strengthened by the inclusion of diverse health care provider groups.

It should be noted that there was no explicit use of behavioural science during the development of both the viral prescription pad and the interview questions for family physicians. This may limit the efficacy of the intervention and quality of data collected in the follow-up physician survey. However, it is also worth noting that the viral prescription pad that was developed by the local ASP contains many of the aspects that are were included in similar documents that were created with the use of behavioural science (see, for example, [41]).

One limitation to the physician interviews is that most family physicians were enlisted from only two urban clinics, and therefore the sample may not be representative of the wider family physician population. This may also provide a potential benefit as it created a more homogeneous population of physicians, which may allow for saturation of data with fewer participants [20]. One of the clinics was academically affiliated, where they have the benefit of having residents contribute to patient education, as well as extra resources for physician education. Also, due to limited data on the rural setting, stratification of data based on location of practice was not possible. Further, it was not possible to stratify the physicians based on years of experience, as these data were not available for all participants. In addition, the opinions do not represent views of physicians who primarily practice in a fee-for-service model or walk-in clinic setting which may again, limit generalizability. Lastly, the interviewees were from clinics that the local ASP had previously worked closely with. Therefore, the uptake of these tools in clinics outside the immediate contact group remains unknown. A major strength of the physician interviews is that the interviewer was not the intervention creator/deliverer, thus reducing the chances of bias against negative disclosure.

The final public survey was limited by the fact that it was only available online. This means that people who did not have access to a mobile device while in one of the local clinics and those who were not connected to one of the social media platforms that were used to distribute the survey may have been unable to provide feedback. Also, due to the nature of sharing the survey link through personal social media platforms, our respondent demographics skew towards a group with a higher than average level of education (89% with post-secondary education, Table 1, versus 25% in Canada [42]). One strength of this study is that the study team was able to receive feedback from people across the country by sharing the survey link via social media, allowing for the potential for feedback from a more diverse population.

## Conclusions

In order to counter the rising rates of antimicrobial resistant infections, it is imperative that health care providers in the community engage in antimicrobial stewardship. Other studies have shown that the use of patient information leaflets during consultations with family physicians for common infections may play a role in reducing unnecessary antibiotic prescriptions [43]. Based on the current study, our local PHC providers are willing to engage in antimicrobial stewardship but many did not feel as though they were equipped with the right tools to help educate both themselves, as well as patients, on the prudent use of antibiotics. When provided with educational tools such as a viral prescription pad, community-based family physicians indicated the utility of this resource in teaching and promoting conversations with patients. Indeed, the general response from the public survey was that this would be a useful tool; this is in line with evidence provided by Bunten and Hawking [41]. However, physicians should be cautious about making assumptions as to patients’ preferences for the method of information delivery. While almost half of the physicians that were interviewed indicated their preference to deliver URTI symptom management information verbally, the data indicate that most patients might prefer to receive this information both verbally and in written format. This is also in line with previously published research [24, 35, 36].

Overall, this study provides valuable information to inform PHC providers and health system administrators about the utility of educational tools (like a viral prescription pad) in engaging both providers and patients in antimicrobial stewardship. This study also provides evidence for the optimal method of use of a viral prescription pad in PHC.

## Supplementary information

**Additional file 1:** Supplemental Figure 1. Viral Prescription Pad The viral prescription pad was designed to fill half an A4-sized sheet. The first version created was for adult patients and was made available in printed pads, online as a fillable PDF via the ASP website, and it was integrated into the electronic medical record of some PHC clinics. The key informational components are: affirmation of a viral infection and indication of the likely length of symptoms, a brief statement about the lack of efficacy of antibiotics for viral infections, a list of options for symptom management, and instructions for when to return to a health care provider.

**Additional file 2:** Supplemental Figure 2. Primary Health Care Provider Survey Questionnaire.

**Additional file 3:** Supplemental Figure 3. Physician Interview Script.

**Additional file 4:** Supplemental Figure 4. General Public Survey Questionnaire.

## Data Availability

The datasets used and/or analyzed during the current study are available from the corresponding author on reasonable request.

## Abbreviations

ASP: Antimicrobial stewardship program
PHC: Primary health care
REDCap: Research electronic database capture
URTI: Upper respiratory tract infection
