# Supplementary material for: The viral prescription pad - a mixed methods study to determine the need for and utility of an educational tool for antimicrobial stewardship in primary health care
Source: BMC Fam Pract. 2020 Feb 22;21:42. doi: 10.1186/s12875-020-01114-z (PMC7035666; doi:10.1186/s12875-020-01114-z)
Supplement: Supplementary file 1 — Additional file 1: Supplemental Figure 1. Viral Prescription Pad The viral prescription pad was designed to fill half an A4-sized sheet. The first version created was for adult patients and was made available in printed pads, online as a fillable PDF via the ASP website, and it was integrated into the electronic medical record of some PHC clinics. The key informational components are: affirmation of a viral infection and indication of the likely length of symptoms, a brief statement about the lack of efficacy of antibiotics for viral infections, a list of options for symptom management, and instructions for when to return to a health care provider. [file 12875_2020_1114_MOESM1_ESM.pdf]

Rx For Adult Patients

Patient Name: ______________________________

Date (mm/dd/yyyy): __________________________

The symptoms you presented with today suggest a **viral infection**.

☐ Viral Upper Respiratory Tract Infection (Common Cold): Lasts 7 to 14 days

☐ Flu: Lasts 7 to 14 days

☐ Acute Pharyngitis (“Sore Throat”): Lasts 5 to 7 days

☐ Acute Bronchitis/”Chest Cold” (Cough): Lasts 2 to 14 days; Cough can last 3 to 4 weeks

☐ Acute Sinusitis (“Sinus Infection”): Lasts 10 to 14 days

☐ Otitis Media (“Middle Ear Infection”): Sharp pain is usually gone in 1 to 3 days

☐ Gastroenteritis (“Stomach Infection”): Lasts 1 to 3 days

You have not been prescribed antibiotics because **antibiotics are not effective in treating viral infections** and may even **cause serious harm**.

When you have a viral infection, it is very important to get plenty of rest and give your body time to fight off the virus. If you follow these instructions, you should feel better soon:

- Rest as much as possible
- Drink plenty of fluids (_____ glasses/day; 1 glass = 1 cup or 250mL)
- Wash your hands frequently
- Take over-the-counter medication (consult with a health care professional):
  - ☐ Acetaminophen (*e.g.*, Tylenol®) for fever and aches
  - ☐ Ibuprofen (*e.g.*, Advil®) for fever and aches
  - ☐ Lozenge (cough candy) for sore throat
  - ☐ Nasal spray (*e.g.*, Salinex®) for nasal stuffiness
  - ☐ Dimenhydrinate (*e.g.*, Gravol®) for nausea
  - ☐ Other: ______________________________

Please return to your prescriber if:

- Symptoms get worse / do not improve in_____day(s)
- You develop a high fever (above _____°C/_____°F)
- Other: ______________________________

Visit our website for more information:

www.rqhealth.ca/asp

____
Prescriber

Saskatchewan Health Authority

Antimicrobial Stewardship Program — Stopping Bugs Saving Drugs
