# Supplementary material for: The viral prescription pad - a mixed methods study to determine the need for and utility of an educational tool for antimicrobial stewardship in primary health care
Source: BMC Fam Pract. 2020 Feb 22;21:42. doi: 10.1186/s12875-020-01114-z (PMC7035666; doi:10.1186/s12875-020-01114-z)
Supplement: Supplementary file 2 — Additional file 2: Supplemental Figure 2. Primary Health Care Provider Survey Questionnaire. [file 12875_2020_1114_MOESM2_ESM.pdf]

PART I: DEMOGRAPHICS

1. What is your gender? [male, female, prefer not to disclose]
2. What is your age?
3. What is your role relating to antimicrobial use? [medical doctor, nurse practitioner, dentist, veterinarian, pharmacist]
4. How long have you been practicing / working in your field?
5. Where do you mainly practice/work? [urban (within the city of Regina), rural (outside the city of Regina), both)
6. What best describes your level of education? Select all that apply. (Different options made available depending on the group being surveyed). [Master's Degree (field), Doctorate (field), MD (specialization), DVM, DDS/DMD, BSP/BScPharm, PharmD, NP, BSN]

PART II: KNOWLEDGE AND ATTITUDES

Please rate your response to the following statements (Strongly Agree, Agree, Neutral, Disagree, Strongly Disagree, Not Applicable):

1. I was familiar with the concept of antimicrobial stewardship before this survey.
2. I believe antimicrobial resistance is an issue of concern in humans/animals.
3. I believe inappropriate use of antimicrobials in humans/animals leads to antimicrobial resistance.
4. I believe inappropriate use of antimicrobials contributes to patient/animal harm (e.g. side effects, toxicity, antimicrobial resistant organisms).
5. Based on this continuum, I believe inappropriate antimicrobial use in health care is mainly driven by…

   [Health Care Professionals -------------------------both equally-----------------------------------Patients]

(an option for “neither” included)

6. Based on this continuum, I believe the majority of inappropriate antimicrobial use occurs in…

[Humans (Health Care) --------------------both equally----------------------------Animals (Agriculture)]

(an option for “neither” included)

7. Tailored for:

Physician/Dentist/Veterinarian/NP – I feel comfortable consulting other health care professionals about an antimicrobial prescription (e.g., indication, dose, route, duration).

Pharmacists/Nurses – I feel comfortable discussing an antimicrobial prescription with other health care professionals when I suspect a misuse (e.g., dose, duration).

8. I believe inappropriate use of antimicrobials in human/animal health care can be significantly reduced through antimicrobial stewardship.

9. I believe decreasing inappropriate antimicrobial use will improve patient care.

10. Omit for Pharmacists/Nurses: I believe an antimicrobial stewardship program may interfere with my practicing autonomy.

11. I believe the public needs more education on the correct use of antimicrobials (through school curriculums, public advertisement campaigns, public health seminars, etc.)

12. I possess or have access to the necessary tools or resources to educate my patients/clients about antimicrobial drugs.

13. What kinds of tools or resources would you find beneficial in educating your patients/clients about antimicrobial drugs? [posters/brochures, viral prescription pads – prescription pads containing non-antibiotic management options for patients who have viral infections (omit for pharmacists), antimicrobial stewardship website, video/audio tools, other]

14. Omit for Pharmacists/Nurses: What kinds of tools would you find beneficial to aid you in making evidence-based decisions about antimicrobials? [antibiogram, lab expertise for consults, pharmacy expertise for consults, infectious disease expertise for consults, other]
15. Omit for Pharmacists/Nurses: I have been pressured to prescribe antimicrobials by a patient/patient's family/client, even after I advise that the condition does not require antimicrobials.
16. I would attend an educational session (e.g., seminar, workshop, online education) that provides further information about antimicrobial stewardship.
17. I believe research for new antimicrobial agents should be publically funded (e.g., government grants).
18. I am willing to promote antimicrobial stewardship initiatives among my colleagues.
