# Supplementary material for: The viral prescription pad - a mixed methods study to determine the need for and utility of an educational tool for antimicrobial stewardship in primary health care
Source: BMC Fam Pract. 2020 Feb 22;21:42. doi: 10.1186/s12875-020-01114-z (PMC7035666; doi:10.1186/s12875-020-01114-z)
Supplement: Supplementary file 3 — Additional file 3: Supplemental Figure 3. Physician Interview Script. [file 12875_2020_1114_MOESM3_ESM.pdf]

1) How often do you access or refer patients to the Antimicrobial Stewardship Program website (www.rqhealth.ca/asp)? If not regularly, why not (are there other resources that you access)? What are the best features of the website and the most useful?

2) How often do you use the viral prescription pad? What features do you like and which are not useful? What would you change, if anything? How does this tool aid in preventing unnecessary antibiotic prescriptions?

3) How often do you use the patient information pamphlet(s)? What do you like/dislike about them? What would you change, if anything? How do they help you in conversations with patients about antibiotic resistance?

4) How does the antimicrobial stewardship commitment poster help you to engage patients in conversations about appropriate antimicrobial use? How does it aid in preventing unnecessary antibiotic prescriptions?

5) How often do you attend (in person/through Telehealth/via WebEx) the Antimicrobial Stewardship Program Education Rounds (second Tuesday of the month at noon)? What kinds of information provided are most useful in your practice?

6) Do you feel the suite of available tools is useful for promoting awareness of antimicrobial resistance and for helping community health-care providers to reduce unnecessary antimicrobial prescriptions?

7) Is there a need to revise or add any new tools? If so which ones?

8) What is the best format(s) for you and your patients to use the tools?

    a. hard copy (print) on the website

    b. e-documents that can be printed or e-mailed to patients

    c. e-documents embedded into the EMR that can be printed or e-mailed to patients

9) Before we wrap things up are there any last comments you have regarding this area of research?
