# Supplementary material for: The viral prescription pad - a mixed methods study to determine the need for and utility of an educational tool for antimicrobial stewardship in primary health care
Source: BMC Fam Pract. 2020 Feb 22;21:42. doi: 10.1186/s12875-020-01114-z (PMC7035666; doi:10.1186/s12875-020-01114-z)
Supplement: Supplementary file 4 — Additional file 4: Supplemental Figure 4. General Public Survey Questionnaire. [file 12875_2020_1114_MOESM4_ESM.pdf]

1. Your age?
2. Your sex? [male, female]
3. Your postal code?
4. What is the highest level of education you have completed? [elementary school, high school, post-secondary (undergraduate degree, technical training, etc.), graduate degree (Masters, PhD)
5. Do you have a regular family doctor or are you more likely to visit a walk-in clinic when seeking care? [I have a family doctor, I generally use a walk-in clinic (or emergency room)]
6. How often do you seek care from a primary care provider when you experience symptoms of an upper respiratory tract infection? (Primary care providers could be doctors, nurse practitioners, pharmacists, etc. Upper respiratory tract infection symptoms can include a cough, runny/stuffy nose, sore throat, ear pain, fever/chills, etc.) [every time, often, sometimes, rarely, never]
7. How would you rate your understanding of the uses of antibiotics? [no understanding, minimal understanding, moderate understanding, good understanding, high level understanding]
8. When you receive care from a primary care provider for an upper respiratory tract infection, do you expect to receive antibiotics? [yes, no]
9. When you receive care from a primary care provider for an upper respiratory tract infection, are you provided with printed information for symptom management? (Printed information could be: hand-written notes, informational pamphlets, a website reference, a viral prescription like the one below, etc.) [always, sometimes, rarely, never]
10. When you receive care from a primary care provider for an upper respiratory tract infection, are you provided with verbal instructions for symptom management? [always, sometimes, rarely, never]
11. When receiving information about symptom management for an upper respiratory tract infection, do you prefer: [printed information, verbal instructions, both printed and verbal

information] An example of printed information for symptom management for viral infections like a cold or the flu provided here.

12. Would you prefer having a handout, like the viral prescription pad above, given to you by your care provider when you are diagnosed with a viral upper respiratory tract infection? [I would prefer receiving this document, I would NOT prefer receiving this document, I find this document useful but would also like receiving verbal instructions from my care provider]
